# Super‐Enhancer Target Gene CBP/p300‐Interacting Transactivator With Glu/Asp‐Rich C‐Terminal Domain, 2 Cooperates With Transcription Factor Forkhead Box J3 to Inhibit Pulmonary Vascular Remodeling

**DOI:** 10.1111/cpr.13817

**Published:** 2025-02-05

**Authors:** Songyue Li, Jingya Zhang, Xu Wang, Xinru Wang, Yuyu Song, Xinyue Song, Xiuli Wang, Weiwei Cao, Chong Zhao, Jing Qi, Xiaodong Zheng, Yan Xing

**Affiliations:** ^1^ Department of Pharmacology Harbin Medical University‐Daqing Daqing Heilongjiang People's Republic of China; ^2^ Central Laboratory Harbin Medical University‐Daqing Daqing Heilongjiang People's Republic of China; ^3^ Department of Pathophysiology Harbin Medical University‐Daqing Daqing Heilongjiang People's Republic of China; ^4^ Department of Pharmaceutical Analysis Harbin Medical University‐Daqing Heilongjiang People's Republic of China; ^5^ Department of Literature Retrieval Harbin Medical University‐Daqing Heilongjiang People's Republic of China; ^6^ Department of Medical Genetics Harbin Medical University‐Daqing Daqing Heilongjiang People's Republic of China

**Keywords:** cell proliferation, pulmonary hypertension, super‐enhancer

## Abstract

The function of super‐enhancers (SEs) in pulmonary hypertension (PH), especially in the proliferation of pulmonary artery smooth muscle cells (PASMCs), is currently unknown. We identified SEs‐targeted genes in PASMCs with chromatin immunoprecipitation (ChIP)‐sequence by H3K27ac antibody and proved that CBP/p300‐interacting transactivator with Glu/Asp‐rich C‐terminal domain, 2 (CITED2) is an SEs‐targeted gene through bioinformatics prediction, ChIP‐PCR, dual‐luciferase reporter gene assays and other experimental methods. We also found that the expression of CITED2 and the transcription factor Forkhead Box J3 (FOXJ3) was reduced in hypoxic mouse PASMCs. In addition, the expression of CITED2 and FOXJ3 also decreased in both the patients with idiopathic pulmonary arterial hypertension (iPAH) and the human PASMCs exposed to hypoxia. The decreased expression of CITED2 was reversed by co‐transfection of FOXJ3 and SEs plasmids. Overexpressing of CITED2 attenuated the PASMCs proliferation induced by hypoxia. Lentiviral overexpression of CITED2 also reversed hypoxia‐induced pulmonary hypertension mice model. Mechanically, the expression of CITED2 by affecting by FOXJ3, which binding with three SEs located in the about 2000 bp of TSS. In conclusion, we first identified that CITED2 is a kind of SEs‐targeted gene, modulated by FOXJ3. The FOXJ3/SEs/CITED2 axis may become a new therapeutic target of PH.

## Graphical abstract

During hypoxia, the transcriptional effect of super‐enhancer in promoting CBP/p300‐interacting transactivator with Glu/Asp‐rich C‐terminal domain, 2 reduced, eventually leading to the thickening of the smooth muscle layer of the pulmonary artery.

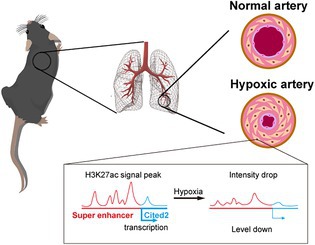

**Abbreviations**
CDK: cyclin‐dependent kinase
CITED2: CBP/p300‐interacting transactivator with Glu/Asp‐rich C‐terminal domain, 2
FOXJ3: Forkhead Box J3
H3K27: lysine 27 of histone H3
iPAH: idiopathic pulmonary arterial hypertension
PAH: pulmonary arterial hypertension
PASMCs: pulmonary artery smooth muscle cells
PCNA: proliferating cell nuclear antigen
PH: pulmonary hypertension
PVR: pulmonary vascular remodelling
SEs: super‐enhancers

## 1 Introduction

Pulmonary hypertension (PH) is a severe cardiopulmonary vascular disease that features isolated elevated pulmonary artery pressure that can eventually lead to right ventricular failure and even death in patients [1]. Hypoxia‐induced PH and pulmonary arterial hypertension (PAH) are the subtypes in PH. Hypoxia is one of the main causes of many diseases [2]. Lung hypoxia can cause massive pneumonia [3, 4], chronic obstructive pulmonary disease [5], bronchial asthma [6], pulmonary fibrosis [7], hypoxic PH [8], and so forth. The main features of PH include progressive pulmonary arteriolar pressure elevation and pulmonary vascular remodelling (PVR) [9]. Although many factors play a role in the progression of PH, the thickening of the smooth muscle layer of pulmonary arterioles due to excessive proliferation of PASMCs, resulting in luminal stenosis and increased blood pressure, is believed to be the main cause of vascular remodelling and right heart failure [8]. However, the underlying regulatory mechanism of PASMCs hyperproliferation in PH remains unknown.

Super‐enhancers (SEs) are large clusters formed by a collection of enhancers with a strong role in regulating gene transcription, and their characteristic is significant H3K27ac modification. In lung cancer, they can use the bridge role of BRD4 to cooperate with a series of transcription factors or transcription activators such as NRF2 to initiate gene transcription and affect cell function [10, 11]. As cis‐regulatory elements, they can cooperate with transcription factors and cofactors to promote the transcription process of target genes [12]. In recent years, the role of SEs in some diseases has been gradually revealed, but research progress on their regulation of gene transcription in PH and their influence on PASMCs function are still slow. In addition, reports of SEs effects on PASMCs proliferation in small pulmonary vessels are limited. SEs can be located upstream, downstream, or within gene promoters; moreover, their distal regulation is much stronger than that of general enhancers, and they can even promote transcription on the promoter by forming a chromatin loop and binding to RNA polymerase II [12]. Limited to a few reports in the field, the relationship between the roles of target genes regulated by SEs and PVR is not yet clear.

CBP/p300‐interacting transactivator with Glu/Asp‐rich C‐terminal domain, 2 (CITED2) is a widely expressed trans‐activator in organisms; it can participate in the transcription and translation process of many genes and may even function as a transcription elongation factor to prolong the transcription of mRNA. Numerous reports describe the regulation of the cell cycle by CITED2 in myeloma cells [13, 14] and liver cancer cells [15]. CITED2 mainly acts as a promoter in many tumour cells [16], and many experiments have shown that CITED2 plays an inhibitory role in the process of cell proliferation [17, 18]. These studies suggest that the role of CITED2 may be condition specific, a phenomenon that may depend on the type of cell or tissue. Here, we screened out the SEs‐targeted gene CITED2 in PH by chromatin immunoprecipitation (ChIP)‐PCR experiments, which may suggest that it plays an important role in PASMCs proliferation and PH progression.

In the present study, we detected attenuated expression of CITED2 and the transcription factor Forkhead Box J3 (FOXJ3) in both hypoxia and SU5416/hypoxia (Su/Hx)‐induced PH models, as well as PASMCs from idiopathic PAH (iPAH) patients [19]. In non‐small cell lung cancer, miRNA inhibits the expression of FOXJ3 by binding to the 3′UTR of FOXJ3, affecting the antiproliferative effect of FOXJ3 [20, 21]. We also determined that FOXJ3 can bind the SEs and promoter region of CITED2 by a bioinformatics prediction method. It is suggested that hypoxia and other PH pathological factors act on the SEs of CITED2 through FOXJ3, affecting the transcription of CITED2 mRNA, and thereby reducing the expression of CITED2.

## 2 Methods

### 2.1 Cell Culture

Cells used in the experiments including mouse pulmonary artery smooth muscle cells (mPASMCs), human PASMCs (hPASMCs), and iPAH‐PASMCs [19]. The culture medium is a kind of special culture medium for smooth muscle cells (SMCM, ScienCell) containing 15% foetal bovine serum (Clark Bioscience, China). The cell density is 5 * 10^6^ inoculated into a 25cm^2^ cell flask. The carbon dioxide concentration is 5%, and the temperature is 37°C humid cultured in an air‐conditioned incubator (Thermo Fisher Scientific). Hypoxia model cell incubator conditions: the oxygen content is 3%, the carbon dioxide content is 5%, the temperature is maintained at 37°C, and the air is humidified. For the cells undergoing gradient hypoxia, the hypoxia time was set to 0, 6, 12, 24, and 48 h, and then the cells were collected to extract protein, and the CITED2 content was detected. JQ‐1 (MCE, 0.5 mM, HY‐13030) and i‐BET (MCE, 5 μg/mL, HY‐163729) was dissolved by DMSO. Normal hPASMCs were purchased from Procell Life Science & Technology Co. Ltd. The cell generations used in the experiment are as follows: third to fifth generation mPASMCs, fourth to sixth generation hPASMCs, and second to fourth generation iPAH‐PASMCs.

### 2.2 Plasmid Overexpression and siRNA Interference

Overexpression plasmid was synthesised by GeneChem Co. Ltd., interference fragments and negative control were synthesised by IBSBIO Co. Ltd. The cells were seeded in 25 cm^2^ culture flasks or well plates and cultured normally for 24 h. Plasmid/interference fragment and negative control were transfected by Lipofectamine 2000 (Invitrogen) according to the instructions. siRNA sequences are listed in Table 1.

**TABLE 1:** Sequences of the siRNA‐CITED2 and siRNA‐FOXJ3.

Name	Sequence‐1 (5′‐3′)
siFOXJ3 sense	GGAAGUGUACAUAGUUAUACA
siFOXJ3 antisense	UAUAACUAUGUACACUUCCAA
siCITED2 sense	CGGCCGCCAGGUUUAACAAUU
siCITED2 antisense	UUGUUAAACCUGGCGGCCGGG
	Sequence‐2 (5′‐3′)
siFOXJ3 sense	CACUGUGACUAACAAAGUAAC
siFOXJ3 antisense	UACUUUGUUAGUCACAGUGUU
siCITED2 sense	AGCACUUCCGAGAUUGCAACC
siCITED2 antisense	UUGCAAUCUCGGAAGUGCUGG
	Sequence‐3 (5′‐3′)
siFOXJ3 sense	GGACCAGGAAGAAGUCCAACA
siFOXJ3 antisense	UUGGACUUCUUCCUGGUCCAG
siCITED2 sense	CCAUAUGUACAGACAAUAAUA
siCITED2 antisense	UUAUUGUCUGUACAUAUGGGG

### 2.3 Western Blot

Cells and tissue were harvested and lysed in RIPA buffer containing protease inhibitors. The cell lysates were separated in a 10% SDS–PAGE Criterion X‐gel (Bio‐Rad) and then transferred to a nitrocellulose filter membrane. The membranes were blocked with 10% skim milk, the primary antibody used include CITED2 (1:500, BOSTER, BM4825); FOXJ3 (1:500, Affinity, AF0605;1:500, Novus, NBP1‐71861); CyA (1:1000, BOSTER, BM1582); CyB (1:1000, BOSTER, BM4667); CyD (1:1000, BOSTER, BM0771); CDK1 (1:1000, BOSTER, PB0561); CDK2 (1:1000, BOSTER, PB0562); CDK4 (1:1000, BOSTER, PB0563); and PCNA (1:1000, BOSTER, BM0104), overnight at 4°C. Anti‐β‐actin antibody (1:1000, ORIGENE) sever as control. After incubation with the secondary antibody, signals from bound antibodies were amplified with ECL (Amersham), and fluorescent bands are presented on X‐ray films and scanned with a scanning machine.

### 2.4 RNA Extraction, Reverse Transcription and Quantitative RT‐qPCR

Total RNA from cells and tissue was extracted with TRizol reagent (Invitrogen) according to the manufacturer's instructions. RNA was reverse transcribed into cDNA using the Reverse Transcription Kit (TOYOBO). Real‐time quantitative PCR was performed with SYBR Green (TOYOBO), and the RT‐qPCR cycling program was as follows: 40 cycles of 95°C for 30 s,60°C for 30 s, and 72°C for 30 s. Relative expression levels were calculated using the comparative ΔΔCt method, and data were normalised to β‐actin mRNA level. The sequences and annealing temperature of primers are listed in Tables 2 and 3.

**TABLE 2:** Sequences of the RT‐qPCR primers for CITED2 and FOXJ3.

Name	Sequence (5′‐3′)
CITED2‐mRNA‐forward	ATCGGCTGTCCCTCTATGTGCTG
CITED2‐mRNA‐reverse	AGTCCTTCCGTCTTTGCGATTTCTG
CITED2‐mRNA‐SE‐428‐forward	CAAGACTGCACGATAGGAAAGGGAAG
CITED2‐mRNA‐SE‐428‐reverse	GAGGTTTTGACGGAGATCAGAAGGG
CITED2‐mRNA‐SE‐429‐forward	GCCATACAGGACGTGCTGCTAC
CITED2‐mRNA‐SE‐429‐reverse	CTGAGTAAGGCTGCTGCTGCTG
CITED2‐mRNA‐SE‐430‐forward	GAGGGCAGGGCAGATCACTAGG
CITED2‐mRNA‐SE‐430‐reverse	AGCATCTACAGGAGAGGAGCAAGG
CITED2‐mRNA‐Promoter‐forward	GAGGGCAGGGCAGATCACTAGG
CITED2‐mRNA‐Promoter‐reverse	AGCATCTACAGGAGAGGAGCAAGG
CITED2‐mRNA‐Desert‐forward	TGGGAGAAACCTCAGGGGACTTC
CITED2‐mRNA‐Desert‐reverse	GCAGTGTGGAAAAGGAAGACTTGAAAC

**TABLE 3:** The annealing temperature of the RT‐qPCR primers for CITED2.

Name	Temperature (°C)
CITED2‐mRNA	61
CITED2‐mRNA‐SE‐428	61
CITED2‐mRNA‐SE‐429	60
CITED2‐mRNA‐SE‐430	62
CITED2‐mRNA‐Promoter	62
CITED2‐mRNA‐Desert	60

### 2.5 Immunofluorescence Staining

Cells cultured in glass bottom dishes need to be fixed with 4% paraformaldehyde at room temperature. Tissue paraffin sections need to be deparaffinised and antigen repair in advance. The cell membrane was permeated with 0.3% TritonX‐100, blocked with 5% goat serum (ZSGB‐BIO), and then the primary antibody was prepared in proportion and incubated at 4°C overnight. On the second day, the primary antibody was discarded and the secondary antibody was incubated under dark conditions. The nuclei were labelled with DAPI, pictured by the photograph with a live‐cell fluorescence microscope.

### 2.6 Animal Models

Preparation of normoxic mice (control group, 6 C57BL mice, male, SPF grade, purchased from Changchun Yisi Experimental Animal Technology Co. Ltd., normoxic FiO2: 21%, following the ethical operation standard of mice. The weight in all groups was about 20–25 g).

Preparation of hypoxia model mice (FiO2: 10%, feeding for 12 days, 21 days, 8 mice in each group).

Prepare SU/HX model mice (Sugen/hypoxia model mice) (subcutaneous injection of Sugen5416 20 mg/kg + hypoxia for 21 days, followed by normoxia for 28 days, 8 mice in each group). After the model was successfully established, the animals were anaesthetised with tribromoethanol (about 250–300 mg/kg, intraperitoneal injection), and euthanasia under deep anaesthesia.

Preparation of CITED2 lentivirus overexpression model mice (C57BL, 8 mice in each group, male, control virus and overexpression virus were synthesised by IBSBIO Co. Ltd) randomly divided into groups: normoxia group, hypoxia group, hypoxia + negative control group and hypoxia + CITED2 overexpression virus group. The mice were treated with hypoxia (FiO2:10%) for 28 days. For the lentivirus treatment group, tribromoethanol was used for light anaesthesia (about 200–240 mg/kg, intraperitoneal injection), the virus titre was calculated according to body weight, inhaled through the nostril, and then placed in the same hypoxia box for 28 days after being stabilised under normoxia for 1 week. The starting point of hypoxia is the same.

The pulmonary artery, heart, lung, and right ventricle (RV) were isolated; total RNA and protein were extracted; and the content of CITED2 was detected by RT‐qPCR.

### 2.7 Small Animal Ultrasound and Right Heart Catheterisation Experiments

The mice (C57BL, SPF grade, purchased from Changchun Yisi Experimental Animal Technology Co. Ltd.) were first anaesthetised with tribromoethanol and then depilated. The cardiac function and lung function of the mice were detected by echocardiography (Vevo 2100 Imaging System, Visualsonics, lnc., USA). After that, the mouse was placed supine on the operating table and fixed, the right jugular vein of the mouse was exposed, and a probe containing a pressure‐sensing device entered the RV from the jugular vein to monitor the right ventricular pressure and recorded dates in real‐time. Euthanasia under deep anaesthesia after the test, remove the heart and other organs, wash with 1× pre‐cooled PBS, isolate the RV, left ventricle (LV), and interventricular septum (S), right ventricular hypertrophy index RV/(LV + S) was calculated. The residual blood of the remaining organs was washed by PBS, and then tissue protein and RNA were extracted.

### 2.8 Haematoxylin and Eosin Staining and Masson Staining

The paraffin lung tissue sections are dewaxed following these steps: First, they are heated for 2 h at 60°C. Then, they are sequentially dewaxed with xylene, anhydrous ethanol, and an ethanol gradient. After this process, any remaining liquid is gently washed away with running water. Next, the sections are stained with haematoxylin and eosin (H&E) according to the instructions provided with the Beyotime H&E staining kit (C0105S). Subsequently, Masson staining is performed using the Solarbio Masson staining kit (G1340). Once the staining is complete, the sections are mounted with neutral resin and photographed.

### 2.9 EdU Fluorescence Experiment

Cells were cultured at the desired density in 24‐well plates with glass bottoms for at least 24 h. After different treatments were taken according to the experiment, a 10 μmol/L EdU reagent was used to label the cells. Discard the medium, add 1 mL 3.7% formaldehyde to each well, and incubate for 15 min. Remove the fixative, cells were washed 2 times with 3% BSA. The cells were incubated with 0.5% TritonX‐100 for 20 min. Click‐iT EdU buffer is diluted with deionised water at a ratio of 1:10. This buffer needs to be prepared and used immediately. Prepare the Click‐iT reaction mixture. Add these ingredients in the order listed in the instructions for the best results. To use the reaction mixture within 15 days of preparation, remove the osmotic buffer and wash the cells again with 3% BSA, then treat the cells with 0.5 mL of the reaction mixture, shaking the plate briefly to ensure that the reaction mixture covers the cells evenly. After incubation in the dark for 30 min, cells were washed, photographed, and analysed using a live‐cell fluorescence microscope.

### 2.10 Cell Counting Kit‐8 Experiment

The cell was countered and seeded into the 96‐well plate (100 μL cell suspension/well) with three replicate wells for each group. When the cells were allowed to adhere to the wall, transfected followed as cultured for 4 h under normoxia, and then gradient hypoxia after changing culture medium. Finally, add 10 μL of CCK8 (Absin, abs50003) solution to each well (no air bubbles left in the well), and incubate the culture plate in the incubator for about 4 h in the dark. The absorbance at 450 nm was measured with a microplate reader.

### 2.11 ChIP Assay

ChIP was performed to quantify the enrichment of FOXJ3 protein and the modification of H3K27ac (ab177178, Abcam) in the promoter and SE region of the CITED2 gene. Cells were subjected to crosslinking by incubation with 1% formaldehyde for 10 min at room temperature, the reaction was quenched by the addition of glycine to a final concentration of 125 mM, after which the cell lysate was subjected to SDS lysis buffer (Beyotime) for preparation of a nuclear extract. Chromatin fragmentation was performed by ultrasonic treatment (BiLon, 650Y) of the cell in 200 μL of SDS lysis buffer to yield an average DNA size of about 300 bp. The sample was centrifugated (14,000*g* for 5 min at 4°C), and final supernatants were mixed with 1800 μL of dilution buffer, 20 μL for input. The diluted samples were incubated with 70 μL protein A + G Agarose for 30 min at 4°C with rotation, the beads were discarded, and 20 μL supernatant was for negative control. The remaining supernatant was incubated with FOXJ3 antibody or H3K27ac antibody overnight at 4°C with rotation (the negative control was treated in the same way, rabbit antibody to IgG). 60 μL protein A + G Agarose was added to every sample to deposit the corresponding compound. Collect the supernatant gently (1000*g* for 1 min at 4°C). Washing with the following solutions in sequence (Low salt immune complex wash buffer, High salt immune complex wash buffer, LiCl immune complex wash buffer, TE buffer). After washing, fresh Elution buffer was added to the sample to elute twice and the supernatant was transferred to the new tube (total 500 μL). Crosslinks were reversed by incubation of the immunoprecipitated for 4 h at 65°C including 20 μL 5 M NaCl (1 μL for input), followed by 1 h at 45°C in 10 μL 5M EDTA plus 20 μL 1 M Tris (pH 6.5) and 1 μL 20 mg/mL proteinase‐K. Genomic DNA was isolated with the use of DNA Purification Kit (Beyotime) and was quantified byRT‐qPCR analysis relative to input DNA. Primer sequences are listed in Table 2.

### 2.12 Dual‐Luciferase Reporter Gene Assay

The artificially synthesised CITED2 promoter region fragments were cloned into pGL3‐reporter (Sangon Biotech). The constructed luciferase reporter plasmids were co‐transfected with FOXJ3 plasmids into mPASMCs. Wild or mutated CITED2‐SE region fragments were cloned into pGL3‐reporter to examine their function. Cells were cultured in an incubator with 5% CO2 and saturated humidity at 37°C. DMEM (Melun) was renewed every 2–3 days. After transfection for 24 h, the cells were lysed. Luciferase activity was measured using GLOMAX MULTI+ Multimode Microplate Detector (E8032, Promega, WI, USA) and Inject System (E7081, Promega, Wisconsin State. USA) based on the instructions of the Dual‐Luciferase Reporter Assay System kit (Beyotime).

### 2.13 Flow Cytometry

The cell cycle was examined by using an Annexin V‐FITC/PI apoptosis detection kit (Biosharp) according to the manufacturer's protocol. After washing gently with PBS, the cell was fixed overnight with 70% ethanol. Incubated with PI staining solution at 37°C for 30 min, flow analysis was performed, and the cell cycle was determined.

### 2.14 In Situ Hybridisation

After the cells of the same model were fixed with paraformaldehyde (Tissue sections need dewaxing first), they were stained with CITED2 probe (BOSTER, Product number MK3868‐m), and the distribution characteristics of cytoplasm and nucleus and subcellular localisation of CITED2 were determined. There were three gene regions of probes against CITED2 mRNA (5′‐ACGCC TTCAA CGCCC TCATG GGCGA GCACA TACAC TACGG‐3′;5′‐ACGGG ACAAA CCAGC ACTTC CGAGA TTGCA ACCCC AAGCA‐3′;5′‐ACTTC GTGTG CAAGC AGCAG CCCAG CAGAG TCAGC TGTTG‐3′) prepared. The signals were detected by Cy3‐conjugated anti‐digoxin. Cell nuclei were counterstained with DAPI, images were obtained on a fluorescent microscope (Nikon, Japan) in the cell imaging system (Tanon, Shanghai).

### 2.15 Statistical Analysis

The data are expressed as the mean ± S.E.M. Statistical analysis was carried out with the GraphPad Prism 9.0 software. Two‐tailed *t* test was used for comparison between the two groups. Multi‐group analysis was tested by the multiple comparisons between ANOVA and Holm–Šídák's, or by the multiple comparisons between ANOVA and Dunnett's as needed. *p* < 0.05 was considered as a statistically significant difference.

## 3 Results

### 3.1 CITED2 Is the Target Gene Regulated by the SEs

**FIGURE 1:**
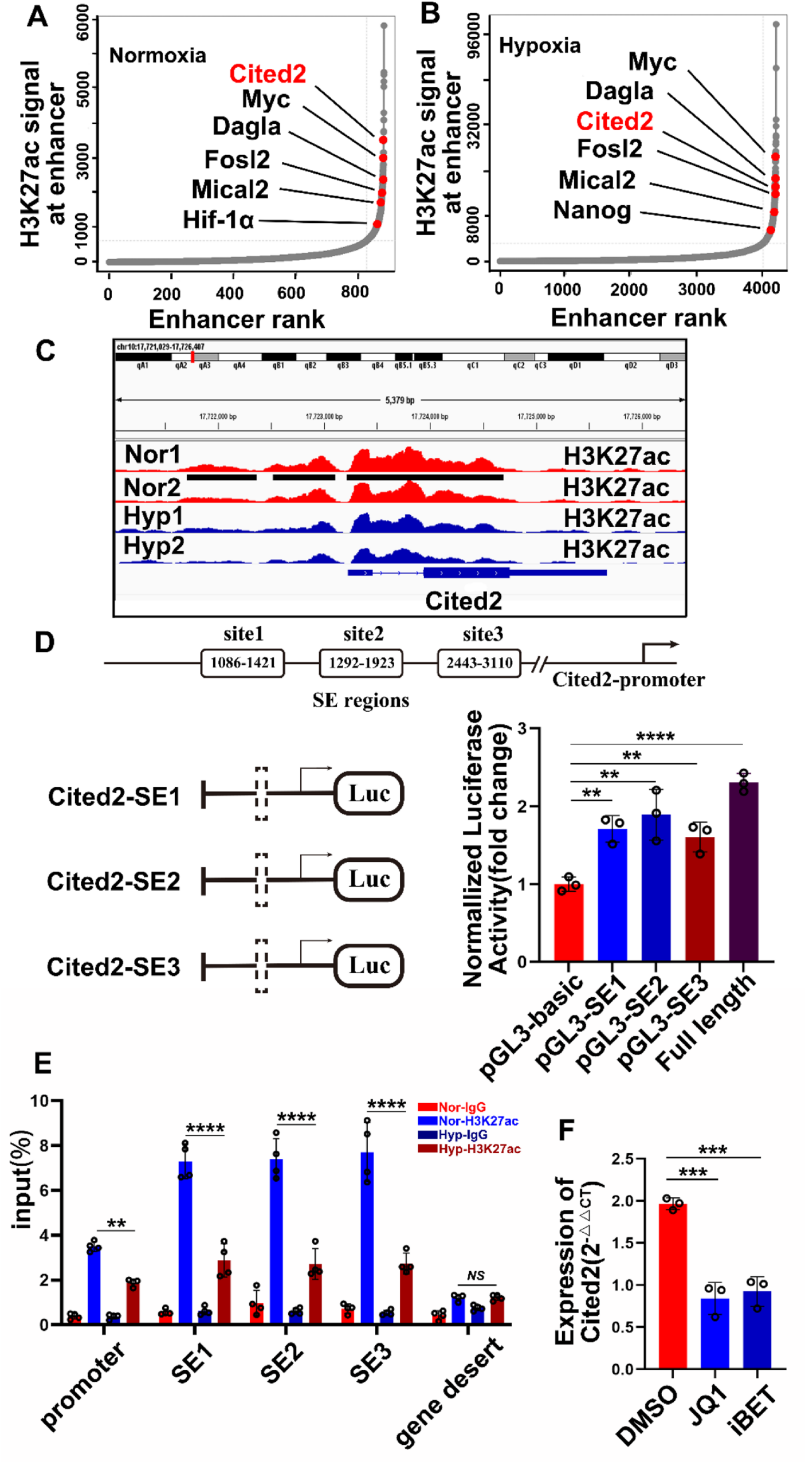
CITED2 is the target gene of SEs. (A, B) The hockey map lists target genes that are regulated by enhancers under normoxia and hypoxia. The entire curve consists of many points, each of which represents a gene. The right side of the image represents genes regulated by SEs. (C) The images represent the level of H3K27ac modification upstream of the CITED2 promoter under normoxia and hypoxia. (D) The three gene fragments and promoter fragments were loaded into plasmids for dual luciferase experiments. In the experimental group with three gene segments, the luciferase activity was higher, and the fluorescence intensity was stronger. (E) The ChIP‐PCR experiment was carried out according to the primers of the promoter constructed by the CITED2 gene and these three gene sequences. The results showed that the region upstream of the CITED2 gene promoter, including the promoter, had significant H3K27ac modification. The fifth primer represented the desert region of the gene. (F) RT‐qPCR results of CITED2 mRNA level after treatment of JQ‐1 and i‐BET. Nor: normal, Hyp: hypoxia, SE: super‐enhancer. (Bar = mean ± S.E.M, *p < 0.05; **p < 0.01; ***p < 0.001; ****p < 0.0001).

In the preliminary preparation, we performed RNA‐seq and ChIP‐seq at the level of mPASMCs cultured in normoxia and hypoxia to screen for downstream genes regulated by SEs. We found that the mRNA levels of 14 genes were downregulated during hypoxia (Supplementary Figure 1C). Moreover, the degree of H3K27ac modification of these genes also decreased significantly (Supplementary Figure 1A,B). Genes modified by H3K27ac under normoxia and hypoxia conditions were screened out. Among them, MYC and NANOG have been reported in PH [22, 23, 24], but there are few related studies on CITED2 (Figure 1A,B), the role of which in PH needs to be clarified. The sequencing results of CITED2 showed three enhanced distinct H3K27ac modification signals near the upstream proximal end of the promoter (Figure 1C). We constructed primers for these three genes and the promoter. ChIP‐PCR results with an antibody against H3K27ac were consistent with the previous conjecture (Figure 1E). We also used SEs inhibitors JQ‐1 and i‐BET to test the effects of SEs to the transcription of CITED2 (Figure 1F). To further verify that these three genes are SE sequences regulating CITED2, we constructed a dual‐luciferase system (Figure 1D). Luciferase activity was significantly increased in the experimental group loaded with these three gene fragments and the CITED2 promoter (Figure 1D), which proved that these three genes are SE sequences.

### 3.2 The Expression Levels of CITED2 Are Downregulated in Hypoxia‐Induced PH

**FIGURE 2:**
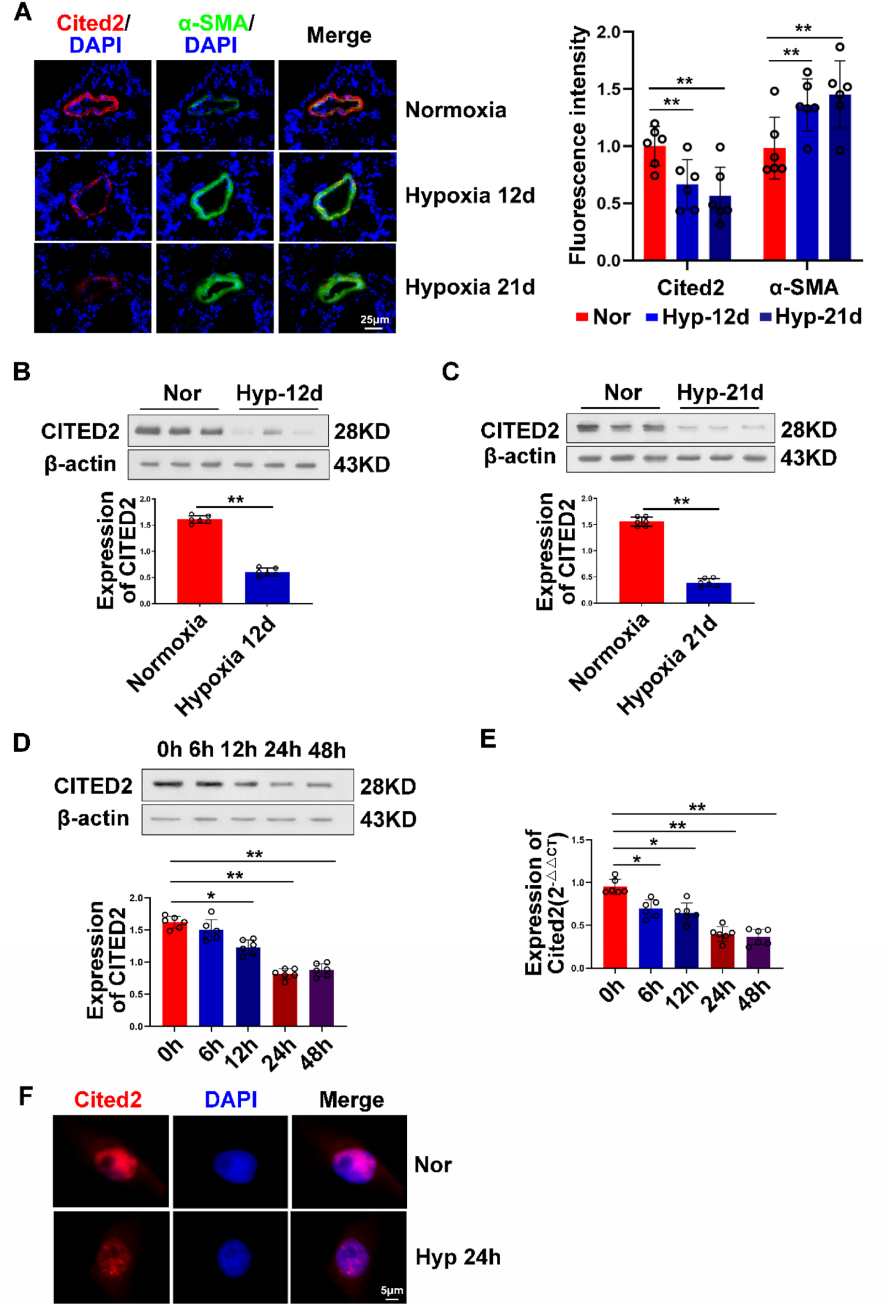
Downregulation of CITED2 in the PH model. (A) The results of the in situ hybridisation experiments showed that the mRNA content of CITED2 was depressed in the pulmonary small vessels of model mice exposed to hypoxia for 12 and 21 days. (B, C) The protein expression of CITED2 decreased in mouse pulmonary arteries on days 12 and 21 of hypoxia. (D, E) The protein and mRNA levels of CITED2 in mPASMCs gradually decreased with increasing hypoxia duration. (F) The result of the FISH demonstrated that CITED2 was depressed in mPASMCs. (Bar = mean ± S.E.M, *p < 0.05; **p < 0.01).

Initially, we conducted experiments using different model samples to explore the expression changes of CITED2 in PH. In the initial investigation, we measured the expression of CITED2 in hypoxic mouse pulmonary artery tissue by in situ hybridisation technology (Figure 2A). The results showed that the mRNA expression of CITED2 in the model group was significantly decreased in the small pulmonary vessels compared with that in the control group. At the same time, by using α‐SMA antibody to localise the smooth muscle layer of cells, we found that the location of CITED2 roughly overlapped with it, which indicated that CITED2 was mainly located in the smooth muscle layer. The in situ hybridisation experiment results of mPASMCs demonstrated that CITED2 was descended (Figure 2F). After that, we extracted the total protein of two hypoxic model mouse lung tissues and found that the protein expression of CITED2 was decreased in the model group (Figure 2B,C). To further test our hypothesis, we evaluated smooth muscle cells from iPAH patients and pulmonary arterioles from SU5416/hypoxia (Su/Hx)‐model mice (Supplementary Figure 2). The results of Western blot and in situ hybridisation experiments showed that the protein and mRNA content of CITED2 in the pulmonary arterioles of SU/HX model mice decreased. In addition, gradient hypoxia was performed at the cellular level, and the content of CITED2 also decreased gradually with increasing hypoxia time (Figure 2D,E).

It is worth mentioning that the expression changes of CITED2 at the cellular and tissue levels showed that CITED2 is significantly decreased in the pulmonary arteries of both the hypoxia and Su/Hx‐induced PH mice models. The results were consistent with those obtained at the cellular level of patients (Figure 8B).

### 3.3 Lentiviral Overexpression of CITED2 Reverses Hypoxia‐Induced PH

**FIGURE 3:**
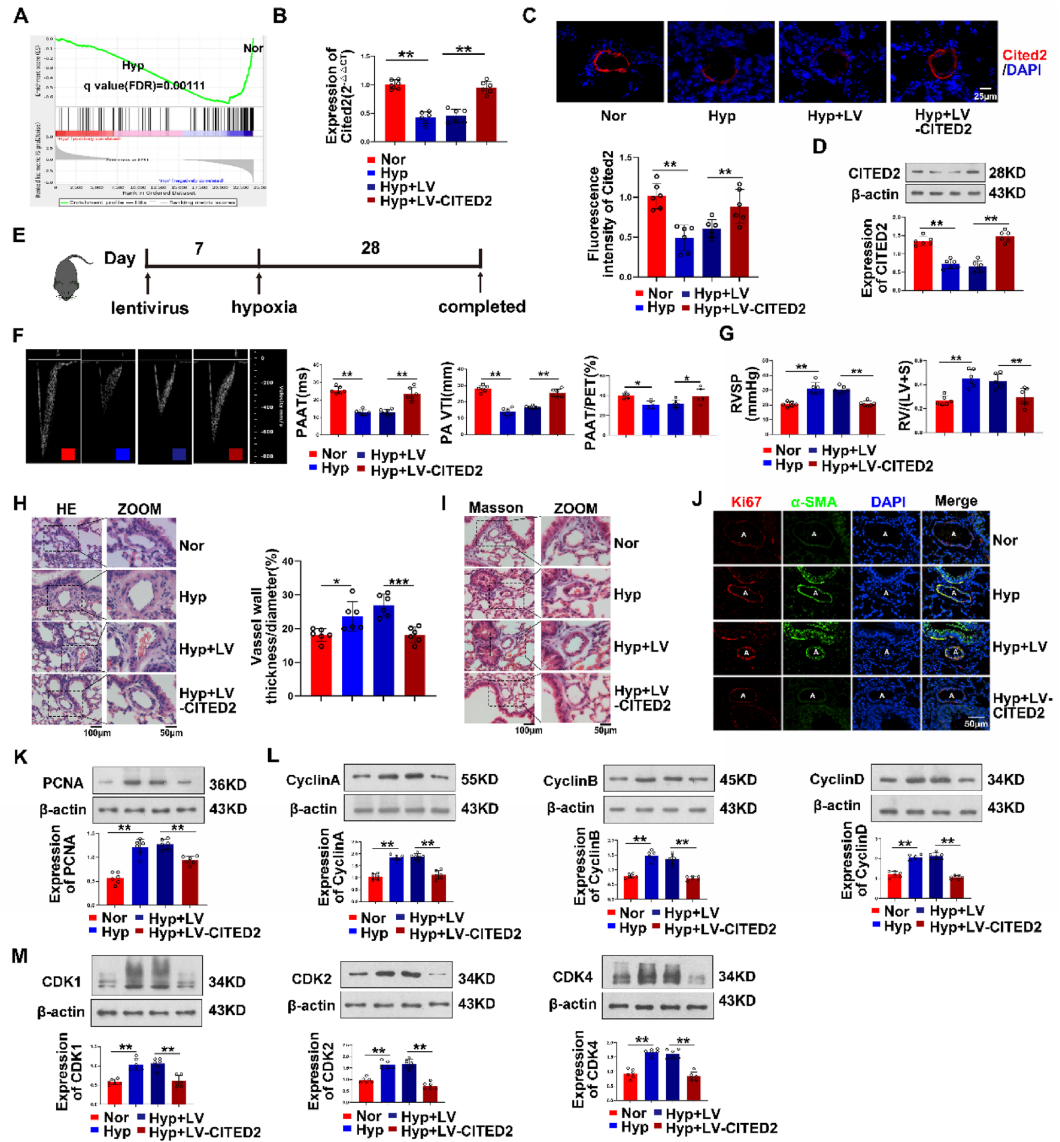
Overexpression of CITED2 at the animal level is able to alleviate PH. (A) The results of gene enrichment analysis in normoxia and hypoxia showed that CITED2 was downregulated in hypoxia, showing a negative correlation. (B–D) Expression of CITED2 mRNA and protein in the pulmonary arteries of model mice. (E) Schematic diagram of lentivirus model mouse construction. (F) The decreased cardiopulmonary function of model mice was effectively reversed by overexpression of CITED2. (G) Right ventricular systolic blood pressure and right ventricular specific gravity were improved in the CITED2 overexpression group. (H, I) H&E and Masson staining of tissue sections of mouse pulmonary arterioles. (K) The expression of PCNA was downregulated in the pulmonary arteries of mice in the lentivirus‐treated group. (J) Tissue immunofluorescence experiments showed that the expression of Ki67 in small pulmonary vessels decreased significantly after CITED2 overexpression. A: artery. (L, M) The expression of cycle‐related proteins in the pulmonary arteries of mice in the lentivirus‐treated group was downregulated. LV: lentivirus. (Bar = mean ± S.E.M, **p < 0.01; ***p < 0.001).

Our KEGG analysis and GO analysis results comprehensively indicated that CITED2 is mainly involved in the cell cycle and cell proliferation process (Supplementary Figure 3). Gene enrichment analysis strongly demonstrated the downregulation of CITED2 in hypoxia (Figure 3A). To further verify the effect of CITED2 on cell proliferation, we first explored its impact on hypoxia‐induced PH at the animal level. The mouse model overexpressed CITED2 by lentivirus through nasal drip for 1 week before hypoxia (Figure 3E). After the modelling was completed (Figure 3B–D), two indirect indicators of PH, right ventricular systolic pressure and right ventricular to left ventricular + spatial weight ratio (RV/(LV + S)), were detected to confirm the success of hypoxia‐induced PH modelling (Figure 3G). In addition, cardiac ultrasound detection showed that hypoxia caused the decline of cardiopulmonary function in mice (Figure 3F). Moreover, the detection results of proliferation‐related indicators in mouse pulmonary arteries showed that the proliferation protein PCNA was downregulated after CITED2 virus treatment (Figure 3K). The results of H&E and Masson staining of lung tissue showed that the thickening of pulmonary vascular media was reduced (Figure 3H,I). The Ki67 index showed that the proliferation of the pulmonary artery smooth muscle layer in the lentivirus‐treated group was significantly reduced (Figure 3J). The expression of cyclin proteins and cyclin‐dependent kinases (CDKs) was decreased (Figure 3L,M). These results indicate that CITED2 also effectively inhibited the proliferation of cells at the animal level.

### 3.4 CITED2 Inhibits the Proliferation of mPASMCs

**FIGURE 4:**
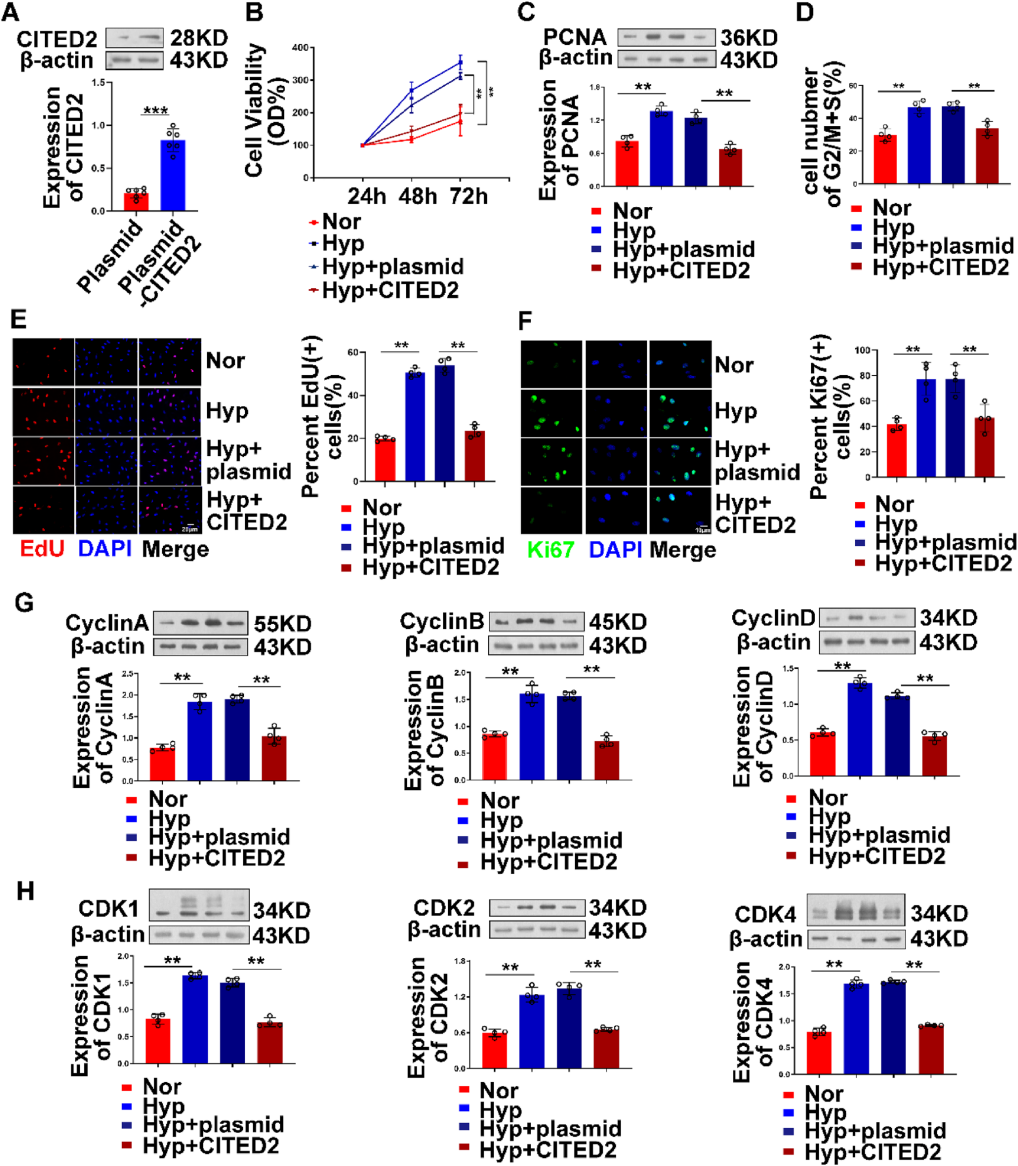
Overexpression of CITED2 can affect the proliferation of smooth muscle cells and hinder cell cycle progression. (A) Western blot results of efficiency detection after overexpression of CITED2. (B) The results of the CCK8 experiment indicated that overexpression of CITED2 could affect the growth and viability of mPASMCs. (C) Overexpression of CITED2 affected the expression of PCNA in cells. (D) After overexpression of CITED2, the proportion of cells in the G2/M and S phase was reduced, which affected cell division. (E, F) The results of the EdU cell fluorescence assay and Ki67 cell fluorescence assay indicated that overexpression of CITED2 could reduce cell proliferation. Red and green represent remarkably proliferating cells. (G, H) Overexpression of CITED2 at the cellular level can affect the expression levels of multiple proteins in the cell cycle. (Bar = mean ± S.E.M, **p < 0.01; ***p < 0.001).

Since the expression of CITED2 is downregulated in hypoxia‐induced PH, we speculated that it may act as a repressor in the development of hypoxia‐induced PH, so we transfected the plasmid into mPASMCs to overexpress CITED2 (Figure 4A), and then, the expression of Ki67 in cells was detected by immunofluorescence assay. Compared with the pure hypoxia group, the expression of Ki67 protein and PCNA was significantly inhibited after overexpression of CITED2 (Figure 4C,F), and the proliferation ability of cells was inhibited. Moreover, the experimental results of EdU and CCK8 further proved our views (Figure 4B,E). Next, we also detected the corresponding cyclin proteins and CDKs to test our hypothesis. The Western blot results showed that overexpression of CITED2 at the cellular level could affect the cell cycle to a certain extent, suggesting that CITED2 inhibits cell proliferation. These experimental results even show that CITED2 has a specific effect on each node of the cell cycle (Figure 4G,H), but the potential mechanism is still unclear. It was found that fewer cells in the CITED2 overexpression group stayed in the G2/M phase (Figure 4D). After that, we repeated the same experiment at the level of hPASMCs, and the final results were consistent with the results obtained at the level of mouse cells (Figure 8), which further confirmed our original hypothesis: CITED2 is involved in cell cycle progression, affecting cyclin proteins and related kinases to reduce cell proliferation. Although the specific mechanism is unknown, it is clear that when the expression of CITED2 decreases, the degree of SMC proliferation increases. When CITED2 is overexpressed, the proliferation process is severely affected, indicating that CITED2 plays an essential role in regulating the cycle progression and proliferation of PASMCs.

### 3.5 The Transcription Factor FOXJ3 Limits the Proliferation of PASMC by Regulating the Transcription of CITED2

**FIGURE 5:**
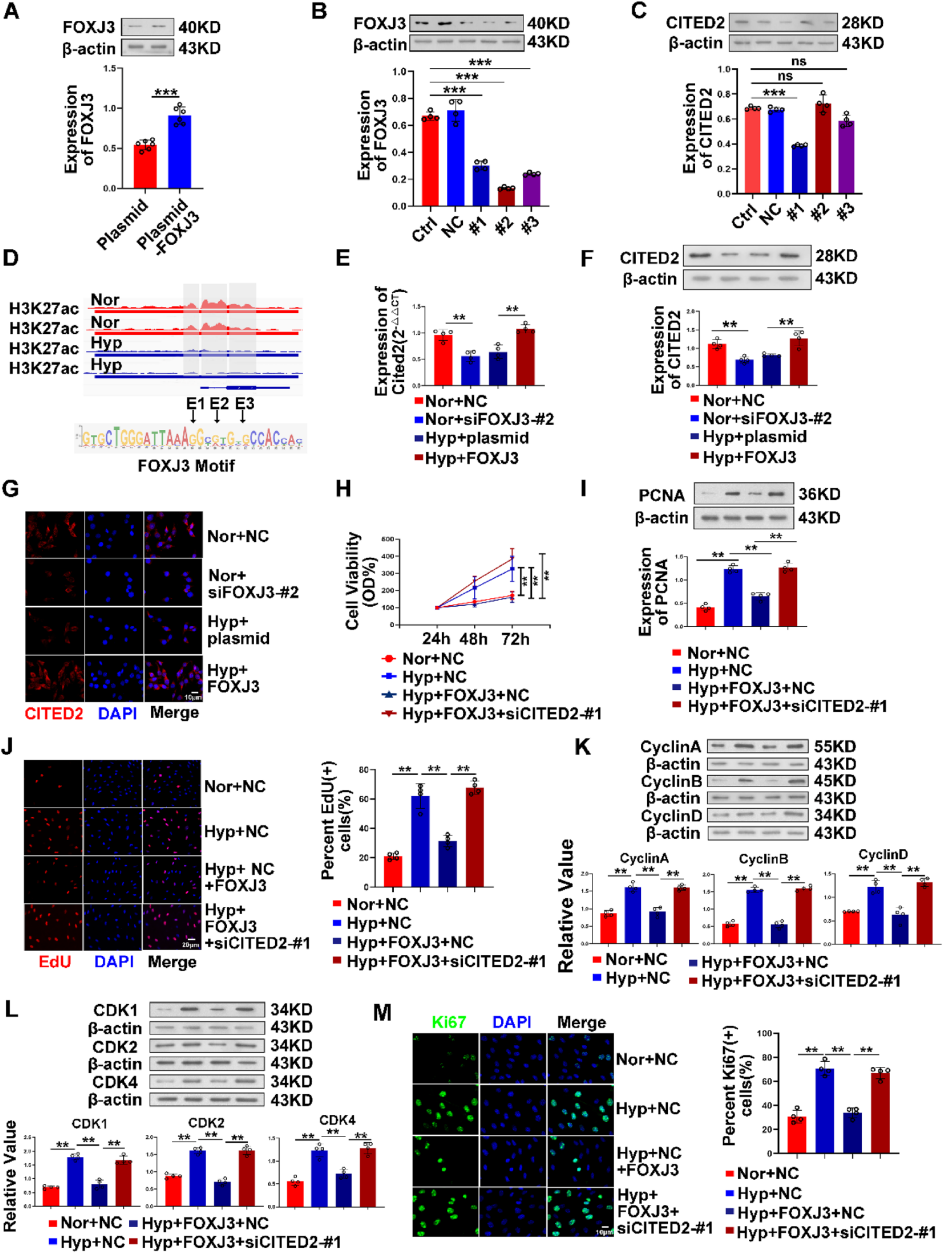
FOXJ3 affects the expression of CITED2 at the transcriptional level and then regulates cell proliferation. (A–C) Western blot results of efficiency detection after overexpression and interference with FOXJ3 and CITED2. (D) Motifs of the transcription factor FOXJ3 predicted from gene motifs by segmenting the CITED2 SEs. (E–G) The mRNA and protein levels of CITED2 were affected by FOXJ3. (H, J) The growth viability of cells decreased after overexpression of FOXJ3 but recovered after interference with CITED2 on this basis. Red in EdU experiments represents cells undergoing proliferation. (I, M) Cell proliferation experiments showed that overexpression of FOXJ3 at the cellular level could affect cell proliferation, and this degree of cell proliferation was restored after interference with CITED2. (K, L) Overexpression of FOXJ3 in mPASMCs decreased the expression of cell cycle‐related proteins and kinase proteins, which was recovered after interference with CITED2. (Bar = mean ± S.E.M, **p < 0.01; ***p < 0.001).

As one protein that regulates the transcription of target genes, transcription factors bind to the promoter of genes when they function in concert with RNA polymerase II and a series of cofactors to mediate expression. Our previous experiments confirmed that CITED2 is an SEs‐targeted gene (Figure 1) and that CITED2 plays an important role in PH. To further screen out the transcription factors that cooperate with SEs, we combined the three SE motifs of CITED2 to predict through the Jaspar website and obtained the gene sequence of the transcription factor FOXJ3 (Figure 5D). From the obtained transcription factor motifs, we constructed FOXJ3 overexpression plasmids (Figure 5A). First, FOXJ3 was overexpressed at the cellular level, and the RT‐qPCR and FISH results indicated that the level of CITED2 was significantly increased (Figure 5E,G). After using siRNA to interfere with FOXJ3 (Figure 5B), the mRNA and protein levels of CITED2 decreased (Figure 5E–G). In addition, the results of CCK8 and EdU experiments also proved that FOXJ3 can affect the growth and proliferation of cells by regulating the expression of CITED2 (Figure 5C,H,J). The results of PCNA (Figure 5I) detected by Western blotting and Ki67 (Figure 5M) agree with this finding. Moreover, cyclin proteins were detected (Figure 5K,L), and the results showed that the expression of CITED2 was affected after the suppression of FOXJ3 and that the proliferation of cells was also inhibited. However, the specific mechanism by which FOXJ3 regulates CITED2 should be further verified.

### 3.6 The Transcription Factor FOXJ3 Cooperates With SEs to Regulate the Expression of CITED2

As cis‐regulatory elements, SEs can participate in the regulation of the transcription of target genes; they can generally be located near the promoter of a gene or far away from upstream or downstream of the promoter. The binding of the SE regions to histones becomes loose when it works, which results in more conducive binding to transcription factors and cofactors. It is now revealed that when SEs are active, the acetylation level of H3K27ac on the histone increases. By detecting the degree of H3K27ac on the histone, it can be judged whether the region is an SE.

**FIGURE 6:**
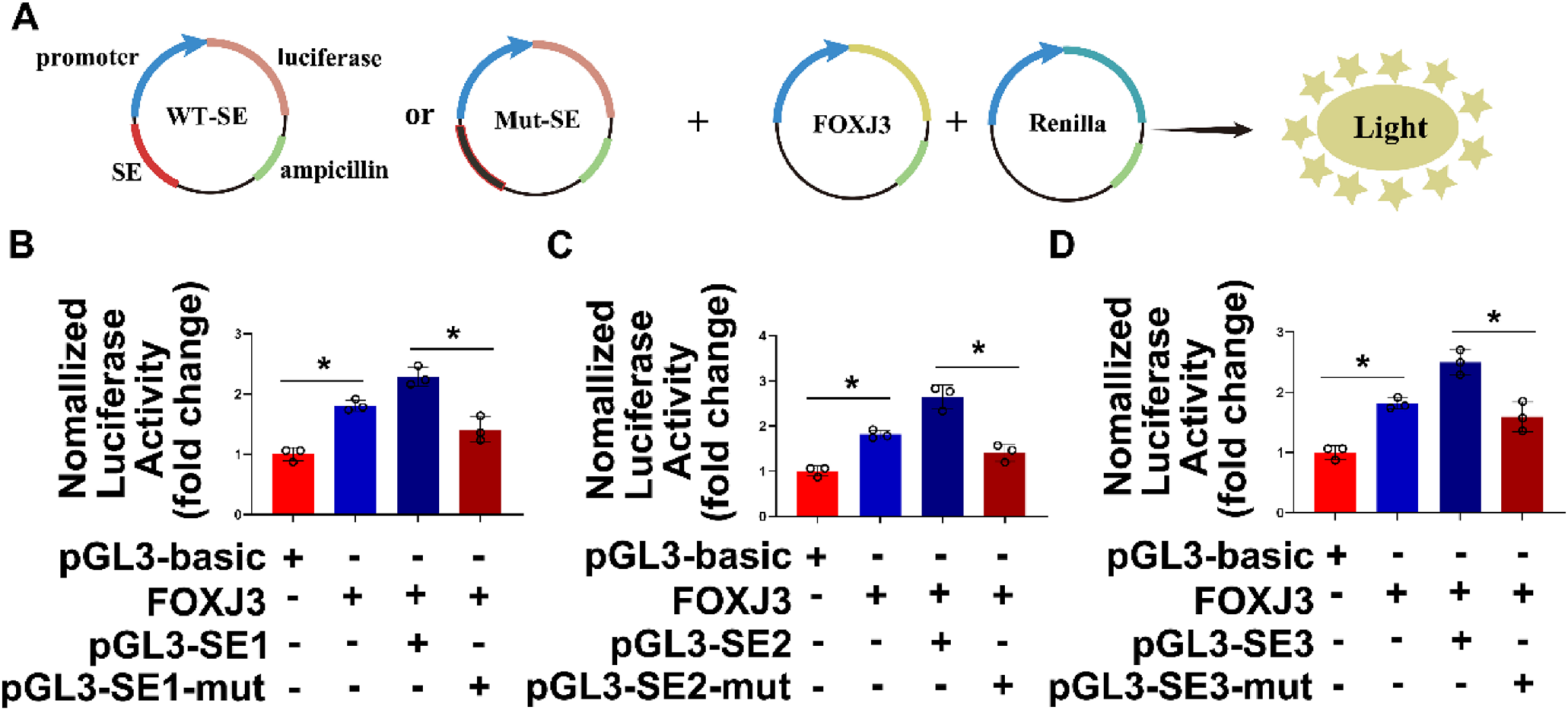
FOXJ3 cooperates with SEs to regulate the transcription of CITED2. (A) Schematic representation of overexpression plasmid construction and transfection. (B–D) In the dual‐luciferase experiment, plasmids containing three SEs motifs were co‐transfected with FOXJ3 plasmids into mPASMCs. When both were present, the luciferase activity was the greatest. (Bar = mean ± S.E.M, *p < 0.05).

The results of co‐screening by ChIP‐seq and RNA‐seq showed that CITED2 is a gene regulated by SEs, and it has been proven that FOXJ3 in the FOX family of transcription factors can regulate the expression of CITED2. We constructed a dual‐luciferase plasmid, loaded the CITED2 promoter with three known SE sequences into the plasmid, and then co‐transfected them with the FOXJ3 plasmid into mPASMCs to verify whether FOXJ3 and SEs regulate the expression of CITED2 (Figure 6A). The results showed that the luciferase activity increased significantly compared with the control group without the SE sequences (Figure 6B–D); after the SEs were mutated, the luciferase activity was reversed. Then, the effect of FOXJ3 on the expression level of CITED2 was verified. FOXJ3 was co‐transfected with the wild type group and the mutant group into mouse cells, and the positive effect of FOXJ3 on CITED2 was more obvious in the presence of SEs. These results strongly demonstrated the effect of FOXJ3 on CITED2 transcription, which is more pronounced in the presence of SEs.

### 3.7 SEs Can Antagonise the Process of Cell Proliferation Under Hypoxia

**FIGURE 7:**
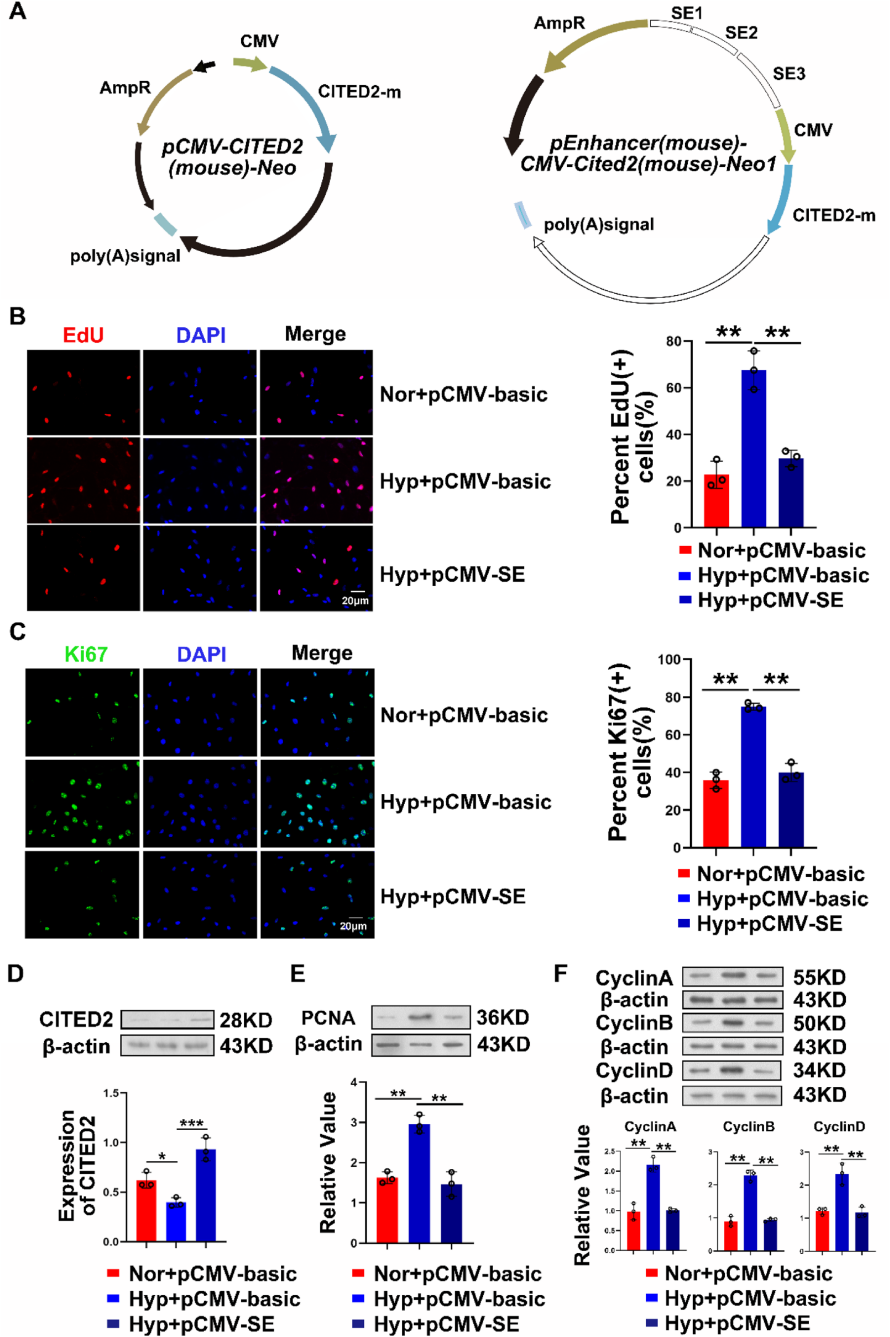
SEs indirectly affect cell proliferation during hypoxia. (A) Schematic diagram of pCMV‐CITED2 and pCMV‐SEs overexpression plasmids. (B, C) The results of EdU and Ki67 immunofluorescence experiments showed that cell proliferation was accelerated under hypoxia and that SEs could indirectly affect cell proliferation by regulating the level of CITED2. Red represents proliferating cells, and green represents Ki67 expression in cells. (D) Western blot results of efficiency detection after overexpression of pCMV‐SEs. (E) Expression of the proliferation indicator PCNA was suppressed in the presence of SEs. (F) When SEs are present, the cell cycle is affected, and cyclin protein expression decreases. (Bar = mean ± S.E.M, *p < 0.05; **p < 0.01; ***p < 0.001).

Previous results have shown that SEs work in concert with FOXJ3, and their effect on the transcription process of CITED2 is positive. To assess the changes in SEs function on downstream effects in hypoxia, we overexpressed a plasmid containing SEs in hypoxic cells (Figure 7A,D). The results of the EdU and Ki67 experiments showed that the effect of SEs was noticeable under hypoxia (Figure 7B,C). In addition to the inhibition of cell proliferation, the proliferation index PCNA also decreased significantly (Figure 7E). Cyclin protein levels were also suppressed after SE overexpression (Figure 7F). This result indicated that rapidly proliferating cells under hypoxia may be indirectly affected by SEs role.

### 3.8 The Therapeutic Effect of CITED2 on Patients With Pulmonary Hypertension

**FIGURE 8:**
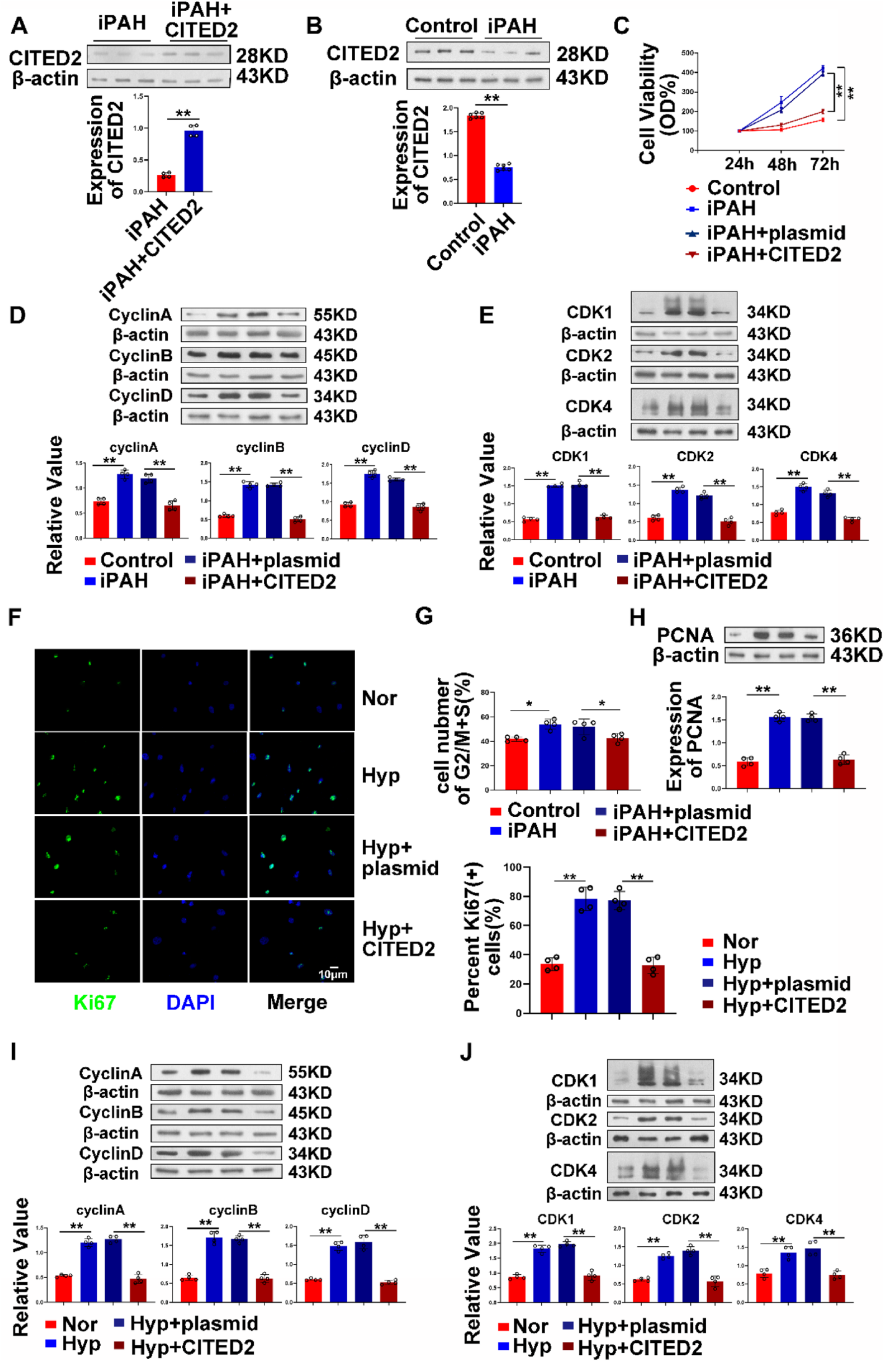
CITED2 can affect the proliferation of idiopathic PAH patient cells. (A) The overexpression efficiency of the CITED2 was detected in iPAH patient cells. (B) Expression changes of CITED2 in iPAH patient cells. (C) The CCK8 experiment showed that overexpression of CITED2 could affect the growth and viability of cells. (D, E) After overexpression of CITED2, the levels of cell cycle‐related proteins decreased in patients. (F) Overexpression of CITED2 in hypoxia‐treated hPASMCs reduced Ki67 levels in the cells. (G) Flow cytometry results showed that overexpression of CITED2 reduced the proportion of cells in the G2/M phase and S phase and affected cell cycle progression. (H) After overexpression of CITED2, the expression of PCNA in patient cells decreased. (I, J) Overexpression of CITED2 in hypoxia‐treated hPASMCs also affected the expression of cell cycle‐related proteins. (Bar = mean ± S.E.M, *p < 0.05; **p < 0.01).

In animals, we examined cells and tissues to confirm the effect of CITED2 on mice mimicking PH. The previous results are sufficient to prove that CITED2 can affect the proliferation of PASMCs in PH, which is beneficial for the disease. Next, we obtained further evidence at the patient level to confirm the therapeutic effect of CITED2. First, CITED2 was overexpressed in cells with iPAH (Figure 8A,B). Western blot analysis showed that the expression of cyclin proteins was decreased (Figure 8D,E), similar to PCNA (Figure 8H). Correspondingly, overexpression of CITED2 also inhibited cell viability (Figure 8C). The same results were obtained by overexpressing CITED2 in normal hPASMCs treated with hypoxia (Figure 8F,I,J). Overexpression of CITED2 can effectively affect the cell cycle of iPAH patient cells. According to the classification and statistics of the cells, CITED2 reduced the number of cells in the G2/M phase and S phase (Figure 8G) and then affected the process of cell division and proliferation. A large number of results have shown that CITED2 plays a role in the process of PH. By inhibiting the proliferation process of PASMCs, CITED2 alleviates the progression of PH. The results given at the animal and patient levels strongly demonstrate the dominant position of CITED2.

## 4 Discussion

**FIGURE 9:**
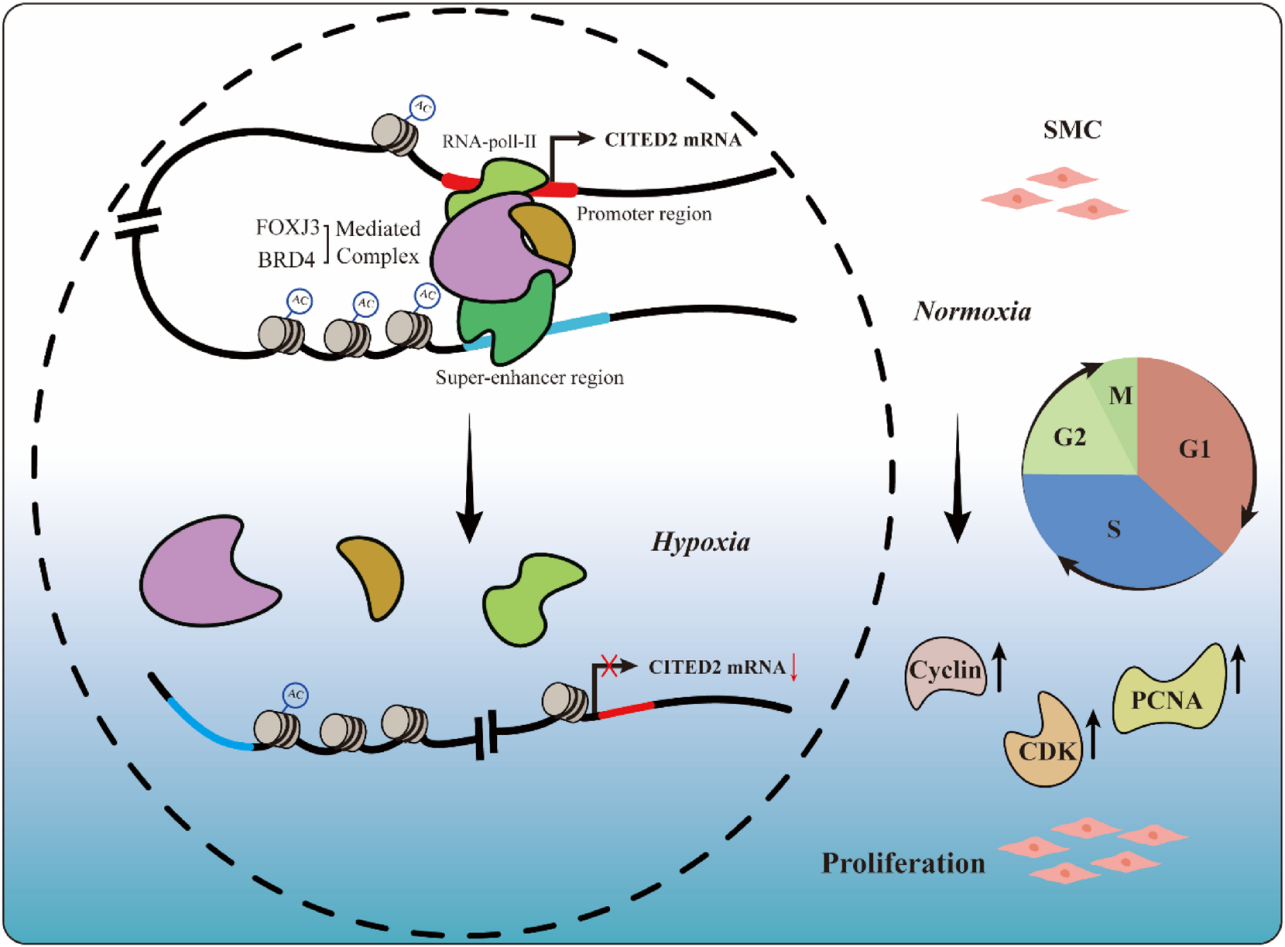
Differential effects of CITED2 on PASMC proliferation under normoxia and hypoxia. Normally, the H3K27ac modification of the SEs and promoter regions of CITED2 is significant. Downstream proteins influenced by CITED2, such as cyclin proteins, CDKs and PCNA, decreased. SMC proliferation is also diminished. However, the phenomenon is reversed under hypoxia.

This is the first study, to our knowledge, to examine the role of CITED2 in the regulation of PASMCs proliferation in PH. Our findings indicate that downregulation of CITED2 plays an essential role in mediated the cell cycle progression and cell proliferation in PH. Furthermore, the expression of CITED2 was modulated by transcription factor FOXJ3, which binding to both the promoter and the SE regions of CITED2. Finally, the overexpressing CITED2 can reverse PVR and PH. Overall, our data suggested that FOXJ3/SEs/CITED2 axis could be considered as a novel strategy in PH therapy (Figure 9).

The increased proliferation and survival of PASMCs is the important feature of PVR during the development of PH. Numerous studies have implicated that CITED2 plays an important role in cell proliferation and the cell cycle progression. In breast cancer cell line, CITED2 is enhanced, promoting MCF‐7 and SKBR‐3 cell proliferation and migration and enhancing their drug resistance [25]. CITED2 gene silencing modulated adherens/tight junction gene expression and reduced cell proliferation in SW480 colorectal cancer cell line [26]. CITED2 knockdown in cell lines with high CITED2 expression led to a decrease of their proliferation, mitochondrial membrane potential, colony formation, and an induced cell cycle arrest and apoptosis in MKN74 gastric cancer cell line [27].

In this study, the overexpression of CITED2 can block the progression of the cell cycle and cause most cells to arrest before the G2/M phase, which affects DNA synthesis and protein synthesis in the S phase. This was consistent with that overexpression of CITED2 significantly suppressed cell growth in HepG2 and BEL7404 hepatocellular carcinoma cell lines, whether CITED2 knockdown significantly increased cell viability and clonogenicity, and promoted G1‐S phase transition in the hepatocyte cell line LO2 and HCC cell line Hep3B [28]. The reason for CITED2 showing different functions in cell proliferation and cell cycle progression can be attributed to its multifaceted role in regulating gene expression and interacting with different molecular pathways. One possible explanation is that CITED2 can act as a coactivator or corepressor of transcription factors, depending on the cellular context and the specific target genes involved. CITED2 has been shown to interact with transcription factors such as HIF‐1α, ATF4, and AP‐1, among others, to promote the expression of genes involved in cell growth and proliferation. On the other hand, CITED2 has also been shown to interact with and inhibit the activity of key cell cycle regulators, such as CDKs and cyclins, which are essential for proper cell cycle progression. Furthermore, CITED2 has been found to interact with various signalling pathways that are important for cell proliferation and cell cycle progression, such as the Wnt/β‐catenin pathway and the TGF‐β signalling pathway. It can modulate the activity of these pathways and influence the expression of target genes involved in cell cycle control. This was confirmed that CITED2 functions as a molecular switch of cytokine‐induced proliferation and quiescence. CITED2 responds to TGF‐α induction and TGF‐β suppression to orchestrate cellular proliferation and quiescence, respectively [29]. Further research is needed to elucidate the specific mechanisms underlying these functions and to better understand the context‐dependent regulation of CITED2 in PASMCs proliferation and cell cycle progression.

Expression of CITED2 can be regulated in different diseases through various mechanisms. It is important to note that the regulation of gene expression is a complex process influenced by multiple factors and can vary depending on the specific context or cell types. The expression of CITED2 can be regulated through genetic alterations or epigenetic modifications. For example, studies have shown that amplification of the CITED2 gene is associated with increased expression in certain types of cancer, including breast cancer [30] and metastatic prostate cancer [31]. Other transcriptional factors or signalling pathways, such as hypoxia‐inducible factors (HIFs) or ETS‐related gene [31] have also been shown to regulate CITED2 expression. These factors can modulate CITED2 expression through direct or indirect interactions with its promoter region or through altering its transcriptional activity. Epigenetic modifications, including DNA methylation and histone modifications, can also regulate CITED2 expression in cancer. Aberrant DNA methylation patterns, especially hypermethylation of the promoter region, can result in gene silencing or reduced expression of CITED2. A study found that a section of the CpG area was easily methylated in the CITED2 promoter region and coding area. After testing the blood samples of children with coronary heart disease, it was found that there were four different types of mutations in the coding region of CITED2, all of which were located in the SGJ region at its carboxyl‐terminal. Studies have shown that gene mutation and increased methylation levels of CITED2 will lead to a decrease in CITED2 expression levels, interfere with its role as a bridge between TFAP2 and CBP/p300, affect the formation of the heart, and lead to various cardiac malformations [32]. Histone acetylation [33] promotes CITED2 expression in gastric cancer cells. In the present study, we first established that CITED2 is an SE‐targeted gene. Under normal circumstances, the expression of CITED2 depends on the regulation of SE with high H3K27ac modification, and this effect is weakened under hypoxia. The sequence of CITED2 SE were found through gene sequencing, which were divided into three segments according to the modified peak of H3K27ac, this is also more conducive to the verification its function in the experiment In addition, our experiment also proved that the three segments of SE have a certain degree of independence in function and regulatory mechanism. The SEs play a critical role in regulating gene expression by recruiting large complexes of transcriptional machinery to specific genes. In disease development, dysregulation of SEs has been implicated in various diseases, including cancer [34], autoimmune disorders [35], and neurodegenerative diseases [36]. Our previously study also confirmed that SE‐associated circular RNA promote PH through modulates endothelial cell injury [37]. The present study found that SEs also involved in the proliferation of PASMCs. Understanding the mechanisms underlying SEs dysregulation may offer new insights into disease mechanisms and potentially lead to the development of targeted therapies in PH.

With the help of bioinformatics prediction, we obtained the transcription factor FOXJ3, which can bind to SEs and CITED2 promoter regions. FOXJ3 belongs to the FOX transcription factor family. Many factors in this family have similar conservative binding sequences. At present, there are many reports about FOXO1 and FOXO3 in this family [38, 39]. Similar to FOXO3, FOXJ3 is mainly used as a transcription factor to regulate the transcription process of the target gene. Although some studies have indicated that FOXJ3 is an important cell cycle regulator that participates in the process of anti‐proliferation [40]. It has been reported that FOXJ3 can combine with the enhancer region of Mef2c to regulate its transcription level [40]. Our research firstly shows that the FOXJ3 participated in PASMCs proliferation through modulated the CITED2 transcription, which is more pronounced in the presence of SEs.

Although not pursued in this study, the mechanisms by which CITED2 induced PASMCs proliferation and cell cycle progression may involve the transcription factor HIF or p53. It is known that HIF plays an important role as a regulatory hub when cells are in hypoxia‐induced PH. After its subunit HIF‐1α enters the nucleus, it binds with HIF‐1β and forms a complex with CBP/p300 to regulate the expression of downstream genes. CITED2 can compete with HIF‐1α for CBP/p300, thus hindering its function [41]. In addition, what we know about the tumour suppressor gene p53 is that the phosphorylation of its N‐terminal residue allows p53 to interact with CBP/p300, resulting in acetylation of the C‐terminal of p53 [42, 43], which can increase the stability of p53. In addition, p53 can activate p21 [44], which can inhibit the activities of CDK and PCNA and block the cell cycle so that it cannot enter the division stage [45]. Acetylation at Lys373 of the p53 protein may lead to stronger binding between p53 and apoptosis‐promoting target genes, thus promoting cell death, while CITED2 can block acetylation at Lys373 of p53 [44, 46]. It has also been reported that the deletion of CITED2 increases the activation of p53 [46], which is jointly activated by CBP/p300. It remains to be determined in future studies whether CITED2 cooperated with HIF1α and/or p53 to induce PASMCs proliferation and cell cycle progression, leading to PVR.

In summary, the present study provides evidence that the FOXJ3/SEs/CITED2 regulatory axis is significantly involved in the hypoxia‐induced PASMCs proliferation and PVR. CITED2may represent a critical component of the signalling pathway linking the PASMCs proliferation and cell cycle progression. SEs are regions of the genome that contain an unusually high density of transcription factor (such as Oct4, FOXJ3) binding sites and are associated with the expression of genes that are important for cell identity and function. Therefore, studies on FOXJ3/SEs/CITED2 will not only enhance our understanding of PASMCs proliferation and cell cycle progression but also help to identify new therapeutic options for the treatment of PH.

## Author Contributions

**Yan Xing** and **Xiaodong Zheng:** conceptualisation, methodology, writing – original draft, supervision, project administration, funding acquisition. **Songyue Li** and **Jingya Zhang:** methodology, validation, formal analysis, writing – original draft. **Xinyue Song**, **Yuyu Song**, **Xu Wang**, and **Xinru Wang:** validation. **Weiwei Cao**, **Xiuli Wang**, and **Chong Zhao:** investigation. **Yan Xin**, **Xiaodong Zheng**, and **Jing Qi:** resources and project administration. All authors are agreed to be accountable for all aspects of the work.

## Ethics Statement

This study and related animal experiments was approved by Ethics Committee of Daqing Campus of Harbin Medical University, and the review number is HMUDQ20230206001. The study was performed in accordance with the Declaration of Helsinki.

## Conflicts of Interest

The authors declare no conflicts of interest.

## Supporting information


**SUPPLEMENTARY FIGURE 1** Downregulated SE‐targeted gene under hypoxia. (A, B) RNA‐seq and ChIP‐seq analysis using mPASMCs cultured in normal and hypoxic 24 h. Antibody against H3K27ac used in ChIP‐seq. Heatmaps depicting differential expression genes under hypoxia(A) and decreased H3K27ac signals under hypoxia(B) separately. (C)78 genes loci with H3K27ac reduced under hypoxia and 1433 downregulated genes under hypoxia. Finally, 14 downregulated genes with diminished H3K27ac signals under hypoxia overlapped.
**SUPPLEMENTARY FIGURE 2**. CITED2 decreased in IPAH and SU/HX model mice. (A) The schematic representation of the SU/HX mode construction. (B) The FISH experiment result showed decreased mRNA level of CITED2 in the smooth muscle layer of pulmonary small vessels of SU/HX mode mice. (C) The protein level of CITED2 in SU/HX mode mice pulmonary artery was diminished. (Bar = Mean ± S.E.M, **P < 0.01).
**SUPPLEMENTARY FIGURE 3**. Genes in hypoxic PH are mainly involved in cell proliferation. (A‐D) In the statistical chart of KEGG and GO analysis results, the top ones are mainly the cellular processes related to cell cycle and proliferation and gene expression regulation.

## Data Availability

RNA‐seq data and ChIP‐seq data that support the findings of this study are available from the corresponding author upon reasonable request.
