# Supplementary material for: Super‐Enhancer Target Gene CBP/p300‐Interacting Transactivator With Glu/Asp‐Rich C‐Terminal Domain, 2 Cooperates With Transcription Factor Forkhead Box J3 to Inhibit Pulmonary Vascular Remodeling
Source: Cell Prolif. 2025 Feb 5;58(11):e13817. doi: 10.1111/cpr.13817 (PMC12584869; doi:10.1111/cpr.13817)
Supplement: Supplementary file 1 — SUPPLEMENTARY FIGURE 1 Downregulated SE‐targeted gene under hypoxia. (A, B) RNA‐seq and ChIP‐seq analysis using mPASMCs cultured in normal and hypoxic 24 h. Antibody against H3K27ac used in ChIP‐seq. Heatmaps depicting differential expression genes under hypoxia(A) and decreased H3K27ac signals under hypoxia(B) separately. (C)78 genes loci with H3K27ac reduced under hypoxia and 1433 downregulated genes under hypoxia. Finally, 14 downregulated genes with diminished H3K27ac signals under hypoxia overlapped. SUPPLEMENTARY FIGURE 2. CITED2 decreased in IPAH and SU/HX model mice. (A) The schematic representation of the SU/HX mode construction. (B) The FISH experiment result showed decreased mRNA level of CITED2 in the smooth muscle layer of pulmonary small vessels of SU/HX mode mice. (C) The protein level of CITED2 in SU/HX mode mice pulmonary artery was diminished. (Bar = Mean ± S.E.M, **P < 0.01). SUPPLEMENTARY FIGURE 3. Genes in hypoxic PH are mainly involved in cell proliferation. (A‐D) In the statistical chart of KEGG and GO analysis results, the top ones are mainly the cellular processes related to cell cycle and proliferation and gene expression regulation. [file CPR-58-e13817-s001.docx]

Supplementary Figure 1. Downregulated SE-targeted gene under hypoxia. (A, B) RNA-seq and ChIP-seq analysis using mPASMCs cultured in normal and hypoxic 24h. Antibody against H3K27ac used in ChIP-seq. Heatmaps depicting differential expression genes under hypoxia(A) and decreased H3K27ac signals under hypoxia(B) separately. (C)78 genes loci with H3K27ac reduced under hypoxia and 1433 downregulated genes under hypoxia. Finally, 14 downregulated genes with diminished H3K27ac signals under hypoxia overlapped.

Supplementary Figure 2. CITED2 decreased in IPAH and SU/HX model mice. (A) The schematic representation of the SU/HX mode construction. (B) The FISH experiment result showed decreased mRNA level of CITED2 in the smooth muscle layer of pulmonary small vessels of SU/HX mode mice. (C) The protein level of CITED2 in SU/HX mode mice pulmonary artery was diminished. (Bar=Mean±S.E.M, **P＜0.01)

Supplementary Figure 3. Genes in hypoxic PH are mainly involved in cell proliferation. (A-D) In the statistical chart of KEGG and GO analysis results, the top ones are mainly the cellular processes related to cell cycle and proliferation and gene expression regulation.

**Supplementary Figure 1**


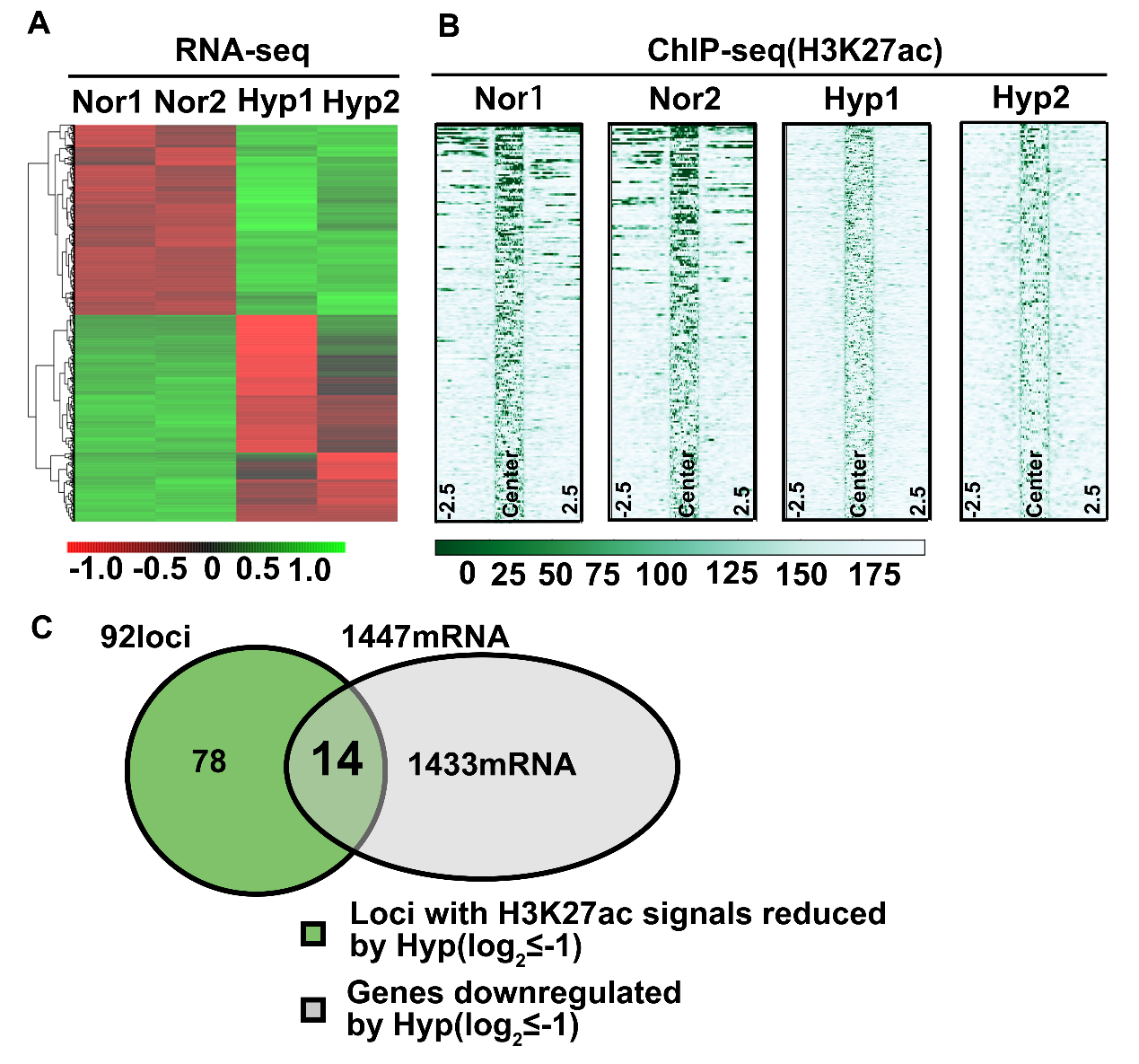


**Supplementary Figure 2**


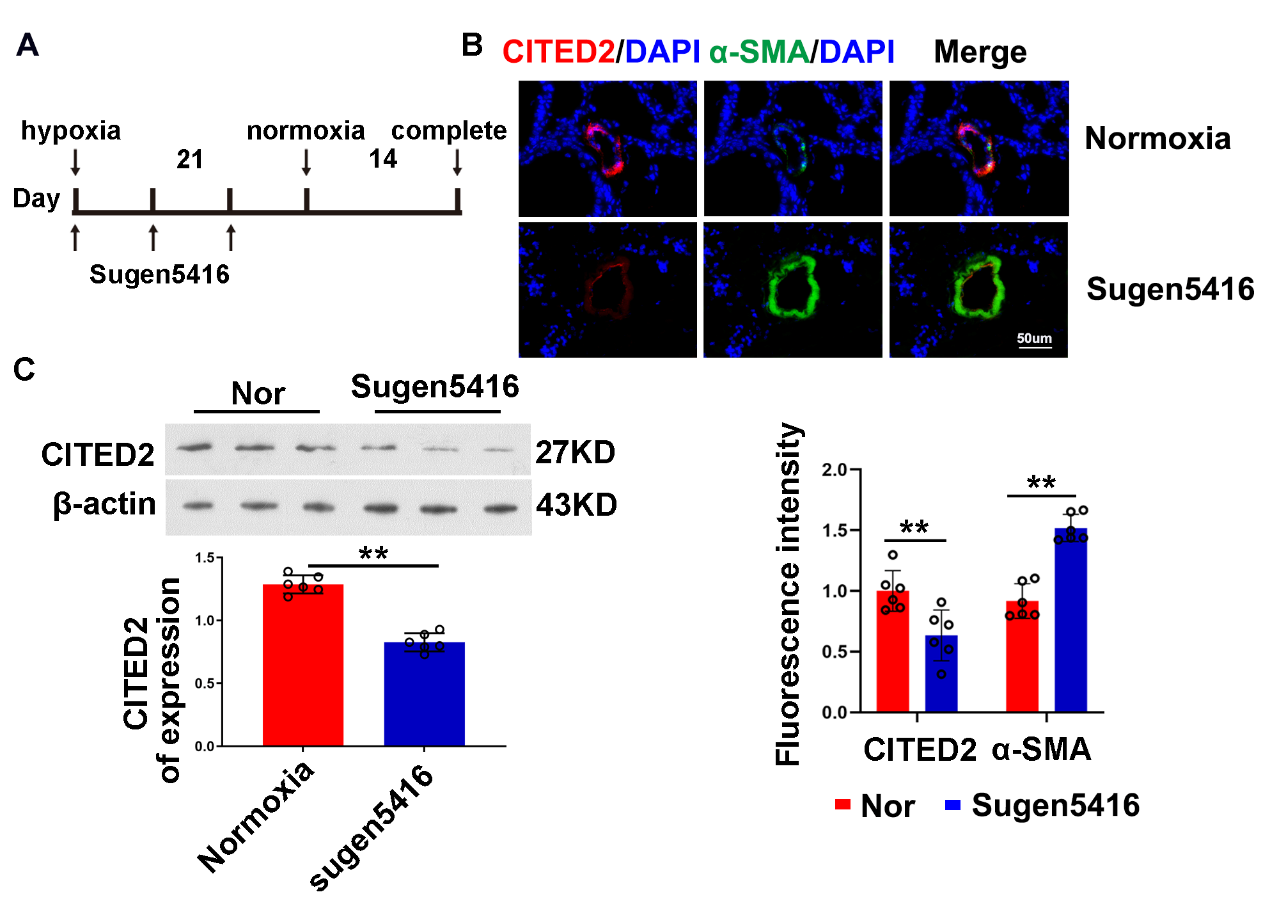


**Supplementary Figure 3**


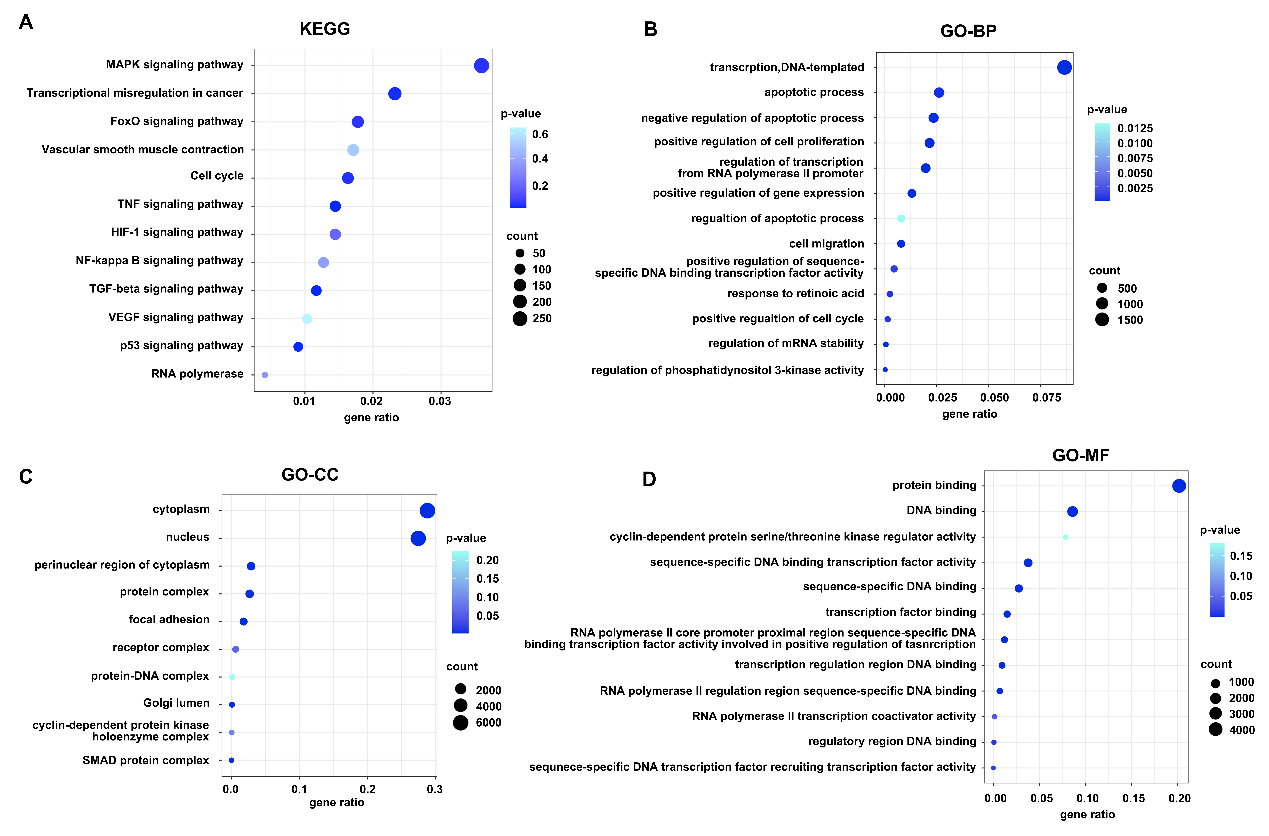


Graphical Abstract

During hypoxia, the transcriptional effect of Super-enhancer in promoting CITED2 reduced, eventually leading to the thickening of the smooth muscle layer of the pulmonary artery.
